# Combined Inositol Hexakisphosphate and Inositol Supplement Consumption Improves Serum Alpha-Amylase Activity and Hematological Parameters in Streptozotocin-Induced Type 2 Diabetic Rats

**DOI:** 10.1155/2019/4143137

**Published:** 2019-10-15

**Authors:** Shadae R. Foster, Lowell L. Dilworth, Jean Sparks, Ruby L. Alexander-Lindo, Felix O. Omoruyi

**Affiliations:** ^1^Department of Basic Medical Sciences, Biochemistry Section, UWI, Mona, Jamaica; ^2^Department of Pathology, UWI, Mona, Jamaica; ^3^Department of Life Sciences, Texas A&M University, Corpus Christi, USA

## Abstract

This study evaluated the effect of combined inositol hexakisphosphate (IP6) and inositol supplement on organ weight, intestinal ATPase activities, complete blood count, and serum analytes in streptozotocin (STZ)-induced type 2 diabetic rats. High-fat diet and a single intraperitoneal injection of streptozotocin (35 mg/kg body weight) were used to induce type 2 diabetes mellitus in Sprague–Dawley rats. The diabetic groups were then treated with either combined IP6 and inositol supplement or glibenclamide for four weeks. Organ weights, intestinal ATPase activities, complete blood count, serum *α*-amylase, total protein, albumin, and globulin content were determined. Pancreatic weight was significantly reduced while relative kidney and liver weights were elevated in the group treated with combined IP6 and inositol supplement compared to the nondiabetic control. Serum *α*-amylase activity for the glibenclamide and combination treated groups was significantly improved compared to that of the untreated diabetic group. Red cell distribution width percentage was significantly lower in the combination treated group compared to that in the untreated diabetic group, while intestinal ATPase activities were unaffected by the treatment regime. Combined IP6 and inositol supplement consumption may protect people with diabetes from increased risk of cardiovascular diseases due to the supplement's ability to maintain red cell distribution width percentage towards the normal control group.

## 1. Introduction

Diabetes mellitus is a chronic disorder that occurs either because of the inability of the pancreas to produce adequate amounts of insulin or its failure to utilize available insulin efficiently. Morbidity and mortality among both youth and adult populations have significantly increased due to diabetes mellitus. There is also a strong association between diabetes mellitus and an increased risk of developing cardiovascular diseases, neuropathies, and microvascular damage to organs including the kidneys and eyes. There are several oral hypoglycemic agents commercially available; however, many have undesirable side effects.

Inositol is a naturally occurring cyclitol formerly referred to as a pseudovitamin. Inositol hexakisphosphate (IP6 or InsP6), also known as phytic acid, is a polyphosphorylated inositol derivative. Both IP6 and inositol are abundantly present in many legumes and whole grains and are present in mammalian cells. They are individually involved in the regulation of insulin secretion [1–4]. Abnormalities in inositol metabolism (inosituria and inositol intracellular depletion) have been observed in several human and animal studies in association with hyperglycemia and insulin resistance. It was proposed that inositol intratissue depletion could contribute to the progression or development of some diabetes-related microvascular complications [5–9]. According to Croze and Soulage, dietary myo-inositol supplementation could reduce and prevent intracellular depletion of inositol in body tissues [10]. Studies have found that myo-inositol supplementation substantially reduced fat accretion and significantly improved insulin sensitivity and glucose tolerance but did not prevent insulin resistance or obesity development in a diet-induced obese (DIO) mouse model [10, 11].

Both IP6 and inositol play vital roles in various cellular processes and are structurally involved in the formation of secondary messengers such as inositol triphosphates (Ins (1, 4, 5) P3 or IP3) and phosphatidylinositol phosphate lipids (PIP2 or PIP3) in mammalian cells. Consequently, metabolic alteration in their levels could affect a wide range of cellular functions. Shamsuddin et al. demonstrated that when IP6 and inositol are combined in the appropriate ratio, the by-products are two IP3 signaling molecules, which are essential cellular regulators [12]. We postulate that one of the several mechanisms by which the combination functions is by acting as a precursor for the formation of lower inositol phosphates and phosphatidylinositols. Treatment with combined IP6/inositol can, therefore, result in the increased cellular concentration of inositol triphosphate. Inositol triphosphate is involved in the regulation Ca^2+^ mobilization and therefore insulin secretion [13, 14]. Previous studies have shown that combined IP6 and inositol possess antidiabetic, anticancer, antioxidant, and organ protective properties and could provide a natural alternative treatment for diabetes mellitus with few or no side effects [15–19]. Hematological and comparative organ weight analyses are widely used to screen drugs and determine toxicity [20]. This study examines the effect of combined IP6 and inositol supplement on organ weights, intestinal ATPase activities, complete blood count, and four serum analytes in streptozotocin-induced type 2 diabetic rats.

## 2. Materials and Methods

### 2.1. Animal Care

Healthy adult male Sprague–Dawley rats (168 ± 5.9 g) were procured from Harlan Laboratories Inc. (Indianapolis, IN, USA). The rats were housed in polypropylene cages with solid floor and bedding materials. The room was maintained at a controlled temperature (22 ± 2°C) with humidity (45 ± 5%) and 12/12-hour light/dark cycle. The animals had free access to standard rat laboratory diet (PicoLab® Rodent Diet 20; 5053) or a high-fat diet containing 45% fat as a percentage of total kcal (D12451; Research Diets Inc., New Brunswick, NJ, USA). They were also provided with clean drinking water *ad libitum*.

### 2.2. Ethical Approval

Study approval was obtained after the review of the protocol by the Institutional Animal Care and Use Committee (IACUC) of the Institute of Biosciences and Technology, Texas A&M Health Sciences Center, Houston, USA (protocol number 011645).

### 2.3. Induction of Type 2 Diabetes

Induction of type 2 diabetes was carried out according to a modified version of previously used protocols [21–23]. The rats were fed 45% high-fat diet for four weeks. At the end of week 2, the rats were administered a single intraperitoneal (i.p.) injection of 35 mg/kg body weight (b.w.) of streptozotocin (Sigma-Aldrich, MO, USA) dissolved in 0.1 M of cold citrate buffer (pH 4.5). This solution was freshly prepared, shielded from light, and administered within 5 minutes of preparation. Control rats were injected with an equivalent volume of citrate buffer alone. One week later, the nonfasting blood glucose level was measured, and animals with a blood glucose concentration ≥300 mg/dL were considered diabetic. To confirm type 2 diabetes, an antidiabetic drug response test was carried out (previously described) [15]. The rats were classified as having type 2 diabetes mellitus based on a positive response to glibenclamide treatment.

### 2.4. Experimental Design

The animal trial was an 8-week study. For the first four weeks, six rats were fed a normal basal diet (PicoLab® Rodent Diet 20; 5053) and 24 rats were fed 45% high-fat diet. Diabetes was induced in 18 of the rats fed with the high-fat diet. At the end of week 4, rats were assigned into the following five groups (six rats per group): nondiabetic control (NC; nondiabetic rats fed basal diets), high-fat control (HFC; high-fat diet and nondiabetic), untreated diabetic control (DC), diabetic rats treated with combined IP6 and inositol supplement (IP6 + INO; 650 mg/kg body weight/day), and glibenclamide positive control group (Glib; 10 mg/kg body weight/day). During weeks 4–8, all rats were fed a basal diet along with their respective treatment regime as outlined. The experiment was designed to administer a dosage of 1% IP6 and inositol combined, which is equivalent to 650 mg/kg body weight at a ratio of 220 : 800. The IP6 and inositol used were extracted from rice and supplied by Vita-Tech International Inc. (Tustin, CA, USA). Glibenclamide is slightly soluble in water. To improve the solubility and bioavailability of the drug, 1% sodium carboxymethyl cellulose (Na-CMC) was used as the transport medium. Combined IP6 and inositol were also dissolved in 1% Na-CMC. Both were administered by oral gavage once daily. The control groups (NC, HFC, and DC) received Na-CMC daily.

### 2.5. Serum and Complete Blood Count Analyses

The rats were fasted overnight and euthanized by decapitation at the end of eight weeks. Blood samples were collected and stored in the appropriate vacutainer tubes. Serum samples were assessed for the concentrations of total protein, albumin, and *α*-amylase using the Stanbio Sirrus Clinical Chemistry Analyzer. The serum globulin measurements were calculated by subtracting the serum albumin concentrations from the serum total protein concentrations. Complete blood count (CBC) analysis was performed on whole blood samples using a Siemens Advia 120 system analyzer.

### 2.6. Organ Weight

After the collection of the blood samples, the liver, kidneys, intestines, heart, spleen, and pancreas were excised and weighed.

### 2.7. Intestinal Analysis

The intestine of each rat, which was free of food materials, was excised and sectioned into proximal (duodenum) and distal (jejunum and ileum) portions. Sodium chloride (0.9%) solution was used to flush the intestinal lumen several times. The scraped mucosa was homogenized and centrifuged (5000 g), and the supernatant was frozen until required for assays [23]. The activities of the ATPases were determined using the methods of Block and Bonting [24] as modified by Bonting et al. [25] and Takeoet al. [26]. ATPase activity was determined by the quantity of inorganic phosphate liberated after incubating with disodium ATP. The protein concentration of the intestinal homogenate was determined using the method of Bradford [27].

### 2.8. Statistical Analysis

All data were analyzed using the statistical package version 20 software (SPSS Inc, Chicago, Illinois, USA). Variation among test groups was evaluated using the one-way ANOVA. *Post hoc* analysis was carried out using Duncan's multiple range test to assess the significant difference among the means (*p* < 0.05). The results were expressed as mean ± S.E.M.

## 3. Results

### 3.1. Organ Weight

Table 1 shows the weights of the organs at the end of the study. There was no significant difference in mean liver weight (*p*=0.078) among the groups. However, the liver weight relative to body weight was significantly increased among the treated and untreated diabetic groups compared to the nondiabetic group. Kidney weight relative to body weight was significantly increased in the treated and untreated diabetic groups compared with the nondiabetic rats (*p* < 0.05). There was a significant reduction in pancreatic weight among the treated and untreated diabetic groups compared with nondiabetic groups. The weights of the spleen and heart among the groups were not significantly different.

### 3.2. Serum Analytes

Serum *α*-amylase activity was significantly lower in the untreated diabetic group compared to the other groups (Table 2). Serum *α*-amylase activity in diabetic rats treated with combined IP6 and inositol or glibenclamide showed a nonsignificant increase compared to the nondiabetic control group. A significant reduction in serum albumin was observed among the diabetic groups compared to the nondiabetic groups (*p*=0.008). Serum total protein or globulin levels showed no significant differences among the groups (*p*=0.461 and *p*=0.675, respectively).

### 3.3. Complete Blood Count Analysis

The data on complete blood count analysis did not show any significant differences in white blood cell (WBC; *p*=0.073), red blood cell (RBC; *p*=0.076) hemoglobin (HGB; *p*=0.106), hematocrit (HCT; *p*=0.072), mean corpuscular volume (MCV; *p*=0.104), mean corpuscular hemoglobin (MCH; *p*=0.253), hemoglobin distribution width (HDW; *p*=0.05), mean corpuscular hemoglobin concentration (MCHC; *p*=0.010), cell hemoglobin concentration mean (CHCM; *p*=0.115), mean platelet volume (MPV; *p*=0.083), and platelet count (PLT; *p*=0.453) among the groups (Table 3). Red cell distribution width (RDW) percentage was significantly elevated in the diabetic control group compared with the nondiabetic control group (*p* < 0.05). Red cell distribution width percentage was maintained in the groups treated with combined IP6 and inositol groups at a level comparable to the nondiabetic control group (*p*=0.106).

### 3.4. Intestinal Mucosa ATPases

**Figure 1:**
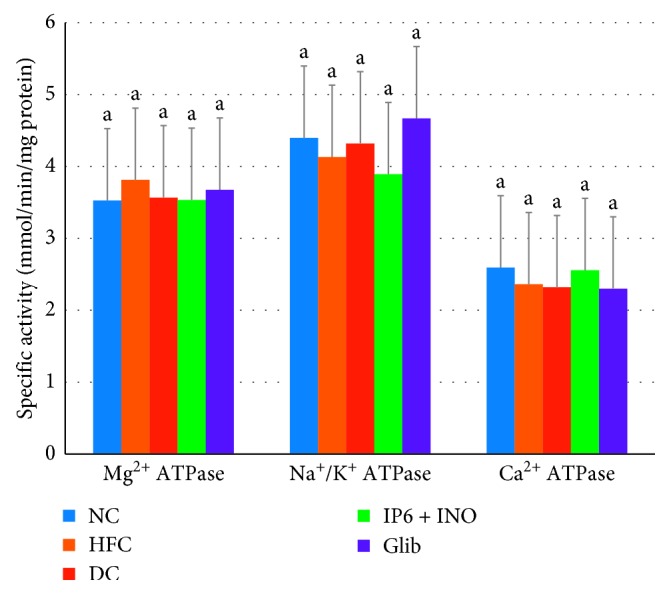
Proximal intestinal ATPase activity among diabetic rats treated with combined IP6 and inositol supplement or glibenclamide. Data are shown as mean ± S.E.M (*n* = 6). The mean values in a column with different superscript letters are significantly different at *p* < 0.05, as assessed by Duncan's multiple range test.

**Figure 2:**
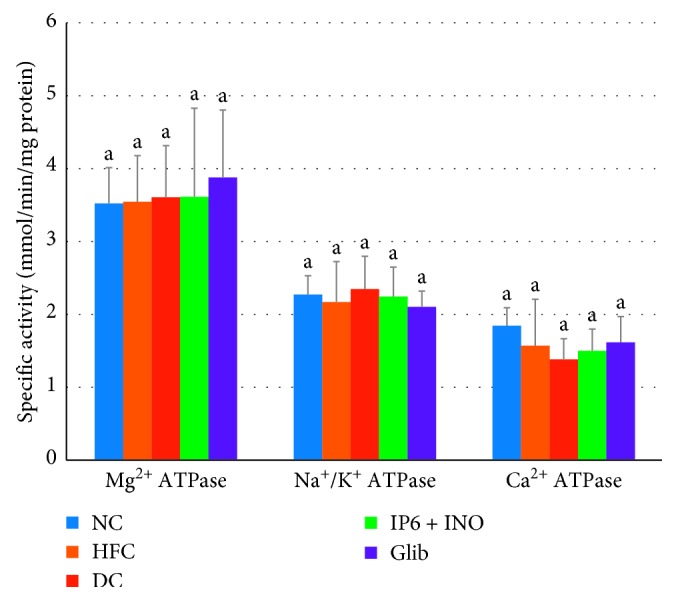
Distal intestinal ATPase activity among diabetic rats treated with combined IP6 and inositol supplement or glibenclamide. Data are shown as mean ± S.E.M (*n* = 6). The mean values in a column with different superscript letters are significantly different at *p* < 0.05, as assessed by Duncan's multiple range test.

The activities of proximal and distal intestinal mucosa ATPases were not significantly altered among the groups (Figures 1 and 2).

## 4. Discussion

Type 2 diabetes is characterized by progressive impairment of insulin sensitivity, followed by beta-cell dysfunction. We have reported earlier that the treatment of STZ-induced type 2 diabetic rats with combined IP6 and inositol resulted in significantly reduced food and fluid intake, fasting blood glucose, serum triglycerides, and total cholesterol and improved insulin sensitivity compared to the diabetic control group thus clearly demonstrating that the combination possesses antidiabetic activities [15]. This present study assessed the association between organ weights of type 2 diabetic rats and those treated with combined IP6 and inositol supplement. The observed significant reduction in pancreatic weight in the treated and untreated diabetic groups compared to nondiabetic groups may be due to the destruction of the pancreatic *β*-cell resulting from the administration of STZ. Previous studies have shown that morphological changes often occur before changes in organ weight [28]. Comparative organ weight analysis between treated and untreated groups of animals is a sensitive and vital endpoint for evaluating the potentially harmful effects of test compounds in conventional toxicology studies [29]. However, there is controversy surrounding the expression of organ weight data relative to the animal's body weight, even though it has become an acceptable practice. It was reported that absolute organ weights, as opposed to relative weights, should be used [30].

Other studies have reported that there is a strong correlation between liver, kidney, spleen, and heart weights in relation to body weights. However, either absolute organ weight or other alternative analysis methods should be utilized when evaluating drug toxicity of other organs such as the pituitary gland, ovaries, thymus, and thyroid-parathyroid [31]. Our data showed no significant differences in the weights of the liver, spleen, and heart among the groups. However, there was a significant increase in relative weights of the liver or kidney in the treated and untreated diabetic groups compared to the normal control group, which may be indicative of liver or kidney hypertrophy. These results are not in agreement with liver morphology, absolute liver weight, and serum markers of liver damage [17]. Serum markers of kidney damage, creatinine, uric acid, and urea nitrogen concentrations indicated that treatment of diabetic rats with combined IP6 and inositol supplement caused no adverse effect on kidney integrity [17]. The serum biochemical markers did not agree with absolute and relative kidney weight which could suggest that relative and absolute kidney weight results are either misleading or significant changes in kidney weight occurred before serum biochemical changes.

The digestive enzyme amylase is synthesized and secreted by the pancreatic exocrine system and salivary glands and is responsible for the metabolic degradation of starch and glycogen to maltose and oligosaccharides. Serum *α*-amylase concentration was significantly lower in the untreated diabetic group compared to the other groups. Reduction in serum *α*-amylase activity observed in diabetic individuals may be due to insulin insufficiency since insulin acts as a secretagogue of amylase when carbohydrate ingestion increases [32, 33]. Previous studies have suggested that low serum *α*-amylase concentration may be due to metabolic abnormalities associated with diminished pancreatic exocrine-endocrine relationship [34, 35]. The observed nonsignificant increase in serum *α*-amylase activity in diabetic rats treated with combined IP6 and inositol or glibenclamide compared to the nondiabetic control group suggest that treatment with the supplement or glibenclamide may restore the metabolic abnormalities associated with an endocrine-exocrine function in diabetes. The observed increasing trend in serum *α*-amylase activity in the treated groups may account for our earlier reported significant reduction in insulin resistance [15].

Albumin, the most abundant serum protein, is a globular protein synthesized exclusively by the liver. Serum albumin levels tend to decrease as chronic liver diseases progress. The two main functions of albumin are the maintenance of plasma oncotic pressure and facilitation of the transport of different metabolites in blood. Protein glycation is the nonenzymatic protein modification that occurs when glucose covalently binds to the protein molecule. Glycation is promoted by elevated blood glucose, as seen in diabetic individuals. Spontaneous modification of the protein molecule will result in the formation of heterogeneous fluorescent molecules called advanced glycation end products (AGEs). The gradual accumulation of AGEs in the blood vessel can lead to many pathological complications in diabetic individuals. One such complication is lipid peroxidation through the generation of free radicals [36]. Low serum albumin is usually associated with cardiovascular risk factors and accelerated atherosclerosis [37]. In this study, treatment with the supplement or glibenclamide did not significantly alter serum total protein, albumin, and globulin levels compared to the untreated diabetic group. A decrease in serum albumin levels in diabetic individuals may not be useful as an early marker for cardiovascular disease development. The prolonged effect of IP6 and inositol combination supplementation on serum protein needs to be further investigated.

Red blood cell (RBCs) count is an important marker in the identification of diabetic patients who are at risk of developing microvascular complications [38]. Red cell distribution width (RDW) is a numerical measure of the amount of variability in the RBC size (e.g., anisocytosis) and is routinely used in the differential diagnosis of anemia [39]. Studies have shown that there is an association between high levels of RDW and the risk of developing cardiovascular disease and nephropathy [40, 41]. The impairment of erythropoiesis has been shown to be related to an increase in RDW which may be due to chronic inflammation and oxidative stress. Inflammation and oxidative stress are cornerstones in the development of type 2 diabetes [42], and they contribute to the development of diabetic complications. Studies have suggested RDW can be used as a predictor of cardiovascular diseases and anemia [43–45]. Weiss and Goodnough reported that inflammation may increase RDW levels by impairing iron metabolism and inhibiting the production or reducing red blood cell survival [46]. On the other hand, Agarwal suggested that oxidative stress may reduce erythrocyte survival through an increase of circulating premature erythrocytes [47]. It was proposed that RDW may be an important clinical marker of vascular complications in individuals with diabetes mellitus. Cakir et al. reported significant increases in RDW values in type 2 diabetic patients [48]. It was proposed that the ideal treatment for diabetes would be a medication that can lower blood glucose as well as reduce oxidation status and diabetic complications [38]. In this study, the RDW values were significantly higher in untreated diabetic rats compared to the other groups. However, red cell distribution width percentage was comparable to the normal control in all the other groups, suggesting that the untreated diabetic control may be at risk of developing vascular diseases. This is plausible, as dyslipidemia which is an established risk factor of cardiovascular diseases was observed in the untreated diabetic group test animals [17]. The observed reduction in RDW levels in the diabetic rats treated with the combination or glibenclamide is indicative of some level of protection against the development of vascular diseases.

Adenosine triphosphatases (Mg^2+^, Na^+^/K^+^ and Ca^2+^ ATPase) have been primarily implicated in the active transport of minerals across the mucosal membrane and energy production. The activities of intestinal mucosa ATPases were not significantly altered among the groups. The observed decreasing trend in the Na^+^/K^+^ ATPase activity in the group treated with combined IP6 and inositol supplement may partly account for its hypoglycemic activity. Studies have shown that reduced ATPase activity may lead to reduced blood glucose concentrations. Dilworth et al. reported that reduction in the absorption of digested materials in rats fed phytic acid extracted from sweet potato might be due to the reduction in intestinal mucosa Na^+^/K^+^ ATPase activity with a subsequent decrease in blood glucose concentrations [49].

## 5. Conclusion

This study demonstrates that combined IP6 and inositol may protect subjects with type 2 diabetes from increased risk of cardiovascular diseases due to its ability to maintain red cell distribution width percentage within the normal range. The combination may also restore the metabolic abnormalities associated with an endocrine-exocrine function in diabetes mellitus.

## Figures and Tables

**Table 1:** Organ weights of diabetic rats treated with combined IP6 and inositol supplement or glibenclamide.

Mean weights	NC	HFC	DC	IP6 + INO	Glib
Liver (g)	10.7 ± 0.31^a^	10.2 ± 0.35^a^	11.1 ± 0.45^a^	11.0 ± 0.37^a^	11.2 ± .19^a^
Relative liver wt. (%)	3.02 ± 0.56^a^	2.78 ± 0.07^a^	3.78 ± 0.13^b^	3.61 ± 0.14^b^	3.38 ± 0.13^b^
Kidney (g)	1.18 ± 0.02^a^	1.2 ± 0.07^a^	1.52 ± 0.09^b^	1.45 ± 0.06^b^	1.44 ± 0.04^b^
Relative kidney wt. (%)	0.33 ± 0.01^a^	0.33 ± 0.01^a^	0.51 ± 0.03^b^	0.47 ± 0.03^b^	0.44 ± 0.03^b^
Spleen (g)	0.67 ± 0.03^a^	0.69 ± 0.06^a^	0.63 ± 0.05^a^	0.60 ± 0.03^a^	0.60 ± 0.03^a^
Heart (g)	1.36 ± 0.06^a^	1.32 ± 0.05^a^	1.30 ± 0.05^a^	1.35 ± 0.51^a^	1.31 ± 0.47^a^
Pancreas (g)	1.33 ± 0.1^a^	1.56 ± 0.1^a^	0.93 ± 0.09^b^	0.97 ± 0.07^b^	0.84 ± 0.06^b^

*Note*. Data are shown as mean ± S.E.M. The mean values in a row with different superscript letters are significantly different at *p* < 0.05, as assessed by Duncan's multiple range test. Relative organ wt.% = (organ weight (g)/body wt (g)) ×100. NC, normal control; HFC, high-fat control; DC, diabetic untreated control; IP6 + INO, combined IP6 and inositol.

**Table 2:** Serum *α*-amylase, total protein, albumin, and globulin concentration in diabetic rats treated with combined IP6 and inositol supplement or glibenclamide.

Parameters	NC	HFC	DC	IP6 + INO	Glib
*α*-Amylase activity (U/L)	1599 ± 21^bc^	1544 ± 57^ab^	1457 ± 31^a^	1689 ± 19^c^	1655 ± 38^bc^
Total protein (g/dL)	7.9 ± 0.16^a^	8.4 ± 0.19^a^	7.8 ± 0.23^a^	7.9 ± 0.26^a^	8 ± 0.26^a^
Albumin (g/dL)	5.13 ± 0.14^a^	4.81 ± 0.16^a^	4.38 ± 0.09^b^	4.5 ± 0.17^b^	4.45 ± 0.12^b^
Globulin (g/dL)	3.1 ± 0.41^a^	3.5 ± 0.31^a^	3.38 ± 0.29^a^	3.28 ± 0.24^a^	3.58 ± 0.26^a^

*Note*. Data are shown as mean ± S.E.M (*n* = 6). The mean values in a row with different superscript letters are significantly different at *p* < 0.05, as assessed by Duncan's multiple range test. NC, normal control; HFC, high-fat control; DC, diabetic untreated control; IP6 + INO, combined IP6 and inositol.

**Table 3:** Complete blood count analysis of diabetic rats treated with combined IP6 and inositol supplement or glibenclamide.

Hematology	NC	HFC	DC	IP6 + INO	Glib
WBC (10^3^/uL)	12.9 ± 0.24^a^	13.42 ± 1.7^a^	9.61 ± 1.27^a^	10.3 ± 0.64^a^	9.7 ± 1.45^a^
RBC (10^6^/uL)	9.45 ± 0.2^a^	9.37 ± 0.3^a^	8.95 ± 0.20^a^	9.71 ± 0.13^a^	9.38 ± 0.2^a^
Hemoglobin (g/dL)	17.23 ± 0.3^a^	17.23 ± 0.4^a^	16.6 ± 0.2^a^	17.62 ± 0.3^a^	17.1 ± 0.47^a^
Hematocrit (%)	51.13 ± 0.3^a^	51.15 ± 1.6^a^	49.8 ± 0.6^a^	53.32 ± 0.6^a^	51.8 ± 1.52^a^
MCV (fL)	54.1 ± 0.64^a^	54.68 ± 1.0^a^	55.8 ± 0.63^a^	54.9 ± 0.23^a^	55.3 ± 0.44^a^
MCH (pg)	18.27 ± 0.1^a^	18.4 ± 0.42^a^	18.6 ± 0.29^a^	18.16 ± 0.2^a^	18.2 ± 0.11^a^
MCHC (g/dL)	33.73 ± 0.1^a^	33.6 ± 0.34^a^	33.4 ± 0.18^a^	33.06 ± 0.3^a^	33.1 ± 0.23^a^
CHCM (g/dL)	33.6 ± 0.9^a^	33.6 ± 0.4^a^	33.4 ± 0.3^a^	32.8 ± 0.3^a^	32.8 ± 0.25^a^
RDW (%)	11.9 ± 0.15^a^	12 ± 0.91^a^	12.9 ± 0.14^b^	12.4 ± 0.17^a^	12.3 ± 0.21^a^
HDW (g/dL)	2.8 ± 0.02^a^	2.8 ± 0.03^a^	2.7 ± 0.06^a^	2.7 ± 0.05^a^	2.61 ± 0.05^a^
PLT (10^3^/uL)	582 ± 143^a^	673 ± 116^a^	637 ± 59.5^a^	681 ± 42.2^a^	685 ± 83.2^a^
MPV (fL)	6.7 ± 0.09^a^	6.4 ± 0.15^a^	6.7 ± 0.15^a^	6.9 ± 0.17^a^	6.7 ± 0.17^a^

*Note*. Data are shown as mean ± S.E.M (*n* = 6). The mean values in a row with different superscript letters are significantly different at *p* < 0.05, as assessed by Duncan's multiple range test. NC, normal control; HFC, high-fat control; DC, diabetic untreated control; IP6 + INO, combined IP6 and inositol; RDW, red cell distribution of width, HDW, hemoglobin distribution width; WBC, white blood cell; RBC, red blood cell; HGB, hemoglobin; HCT, haematocrit; MCV, mean corpuscular volume; MCH, mean corpuscular hemoglobin; MCHC, mean corpuscular hemoglobin concentration; CHCM, cell hemoglobin concentration mean; MPV, mean platelet volume; PLT, platelet count.

## Data Availability

The data used to support the findings of this study are available from the corresponding author upon request.
